# Agents Affecting the Plant Functional Traits in National Soil and Water Conservation Demonstration Park (China)

**DOI:** 10.3390/plants11212891

**Published:** 2022-10-28

**Authors:** Gaohui Duan, Zhongming Wen, Wei Xue, Yuankun Bu, Jinxin Lu, Bojin Wen, Boheng Wang, Sihui Chen

**Affiliations:** 1 College of Grassland Agriculture, Northwest A&F University, Yangling 712100, China; 2 Institute of Soil and Water Conservation, Chinese Academy of Sciences & Ministry of Water Resources, Yangling 712100, China; 3 State Key Laboratory of Soil Erosion and Dryland Farming on the Loess Plateau, Northwest A&F University, Yangling 712100, China; 4 College of Forestry, Northwest A&F University, Yangling 712100, China; 5 East China Survey and Planning Institute of National Forest and Grassland Administration, Hangzhou 310019, China

**Keywords:** plant functional traits, plants diversity, soil properties, random forest algorithm, PLS-SEM

## Abstract

Plant functional traits (PFTs) can reflect the response of plants to environment, objectively expressing the adaptability of plants to the external environment. In previous studies, various relationships between various abiotic factors and PFTs have been reported. However, how these factors work together to influence PFTs is not clear. This study attempted to quantify the effects of topographic conditions, soil factors and vegetation structure on PFTs. Four categories of variables were represented using 29 variables collected from 171 herb plots of 57 sites (from different topographic and various herb types) in Xindian SWDP. The partial least squares structural equation modeling showed that the topographic conditions and soil properties also have a direct effect on plant functional traits. Among the topographic conditions, slope (SLO) has the biggest weight of 0.629, indicating that SLO contributed the most to plant functional traits and vegetation structure. Among soil properties, maximum water capacity (MWC) contributes the most and is followed by soil water content (SWC), weighted at 0.588 and 0.416, respectively. In a word, the research provides new points into the quantification of the correlation between different drivers that may be important for understanding the mechanisms of resource utilization, competition and adaptation to the environment during plant recovery.

## 1. Introduction

The Soil and Water Conservation Demonstration Park (SWDP) is a major innovation of soil and water conservation in China. The ecosystem functions of SWDP include soil and water loss prevention, climate improvement, resource protection, etc., however, with the progress of the times, the goal of SWDP is not only limited to the improvement of park ecology through planting vegetation and construction projects but also plant restoration should become the focus of research. In order to better understand the role of vegetation in soil and water conservation projects, the changes of plant functional traits should not be ignored. The construction of Xindian SWDP can be traced back to 1952. After 68 years of continuous management, the vegetation coverage of the park has recovered from 5% to the current 75% and great changes have taken place in the ecology of the park during the 68 years of continuous management. Therefore, the study of plant functional traits reflecting the ecosystem change strategy is very important for soil and water conservation and restoration development.

As the largest terrestrial ecosystem, grassland ecosystem mainly distributes in ecologically fragile areas due to their special functional traits and strategies [1]. Functional traits are biological attributes that directly or indirectly affect species fitness and endow plants with high adaptability to environmental changes [2,3]. Functional trait variability allows plants to minimize their building costs and maximize functional efficiency. Therefore, to ascertain plant functioning and their ecological strategies in soil and water conservation measures, it is critical to explore how functional traits vary across different measures [4], especially relevant under the ongoing climatic change scenario. For instance, Scots pine (*Pinus sylvestris* L.) has the ability to adjust its leaf/sapwood area ratio, leaf-specific hydraulic conductivity and total leaf area in response to drought [5].

The functional traits of grassland communities vary greatly due to latent influencing factors, such as topographic conditions, soil properties and plant diversity [6]. On the one hand, habitat heterogeneity, due to changes in environmental factors and topography, will lead to the differences in grassland composition [7]. For example, temperature, humidity and altitude will affect grassland vegetation population and change functional traits [8]. On the other hand, there is a complex relationship between soil properties and plant. Soil which controls water and nutrients is the most critical condition for plant growth [9,10]. Soil water content is also considered to be the main factor in determining the composition of grassland vegetation [11]. Lush plants also feed back to soil nutrients and avoid soil erosion through their leaves and roots [12,13]. In addition, high vegetation coverage will create a microclimate through changes to the environment temperature and humidity and then affect soil properties [14]. In recent years, correlation or causation was explained among plant functional traits and the influencing factors. However, how these factors work together to influence plant functional traits and how they interact with each other remains unclear [15]. Hence, to resolve this major problem, the research should quantify the factors that impact on plant functional traits.

Four categories of variables were represented using 29 different observational variables collected from 171 plots of 57 sites in Xindian National Soil and Water Conservation Demonstration Park. We used random forest (RF) algorithm to identify critical indicators of plant functional traits. The RF is a machine learning classification method that has been demonstrated as an efficient algorithm to obtain key factors [16]. Moreover, structural equation modeling (SEM) was used to quantify the influence of these factors on plant functional traits [17]. In this research, we employed the partial least squares structural equation modeling (PLS-SEM). By applying these methods, the main objectives of this study were as follows: (a) Select and quantify appropriate plant functional traits in the study area; (b) Discuss the interaction between topographic conditions, soil properties and plant diversity affecting plant functional traits; (c) Evaluate the effects of topographic conditions and diversity indexes on plant functional traits. The study on the above issues is helpful to understand the status quo and change rules of plant functional traits in the special ecosystem of Xindian National Soil and Water Conservation Demonstration Park; it is of great significance to understand the mechanism of plant community construction under the interaction of different factors.

## 2. Materials and Methods

### 2.1. Study Area

Xindian Soil and WATER Conservation Demonstration Park, located on the left bank of the middle reaches of Wuding River, was built in 1952. It covers an area of 1.44 km^2^ and is all composed of hills. The terrain is very broken, with 31 gullies that are 200 m long and cultivated land above 25 degrees accounting for 49% of the total cultivated land area (Figure S1). According to the observed data in 1952, the annual average soil loss amount was 19,900 t. Since 1952, 24 silt DAMS have been built in the demonstration park. At present, the control degree (through engineering measures and vegetation engineering) of the demonstration park has reached 80%, the forest and grass coverage rate has promoted from 5% to 75% and the sand blocking rate has reached 98%.

**Figure 1:**
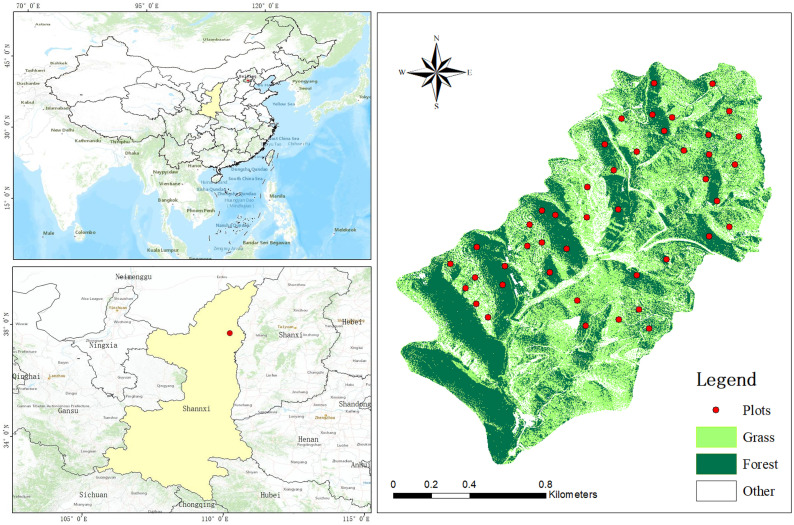
Research area.

The soil texture is sandy loam with dense gullies, which has typical loess hilly and gully landform (Figure 1). It is a temperate continental semi-arid climate, with a monthly mean temperature, ranging from −7.5° in January to 24° in July, mean annual temperature is 8.3° and the mean annual precipitation is 486 mm from 2010 to 2020, most of which occurs in the form of rainstorms from July to September [18].

The study area is dominated by grassland and accounts for more than 80% of the total vegetation area. Our research plots are located at an altitude of 850 m to 1287 m, with a slope range from 3 °C to 40 °C.

### 2.2. Plot Survey, Sample Collection and Analysis

**Figure 2:**
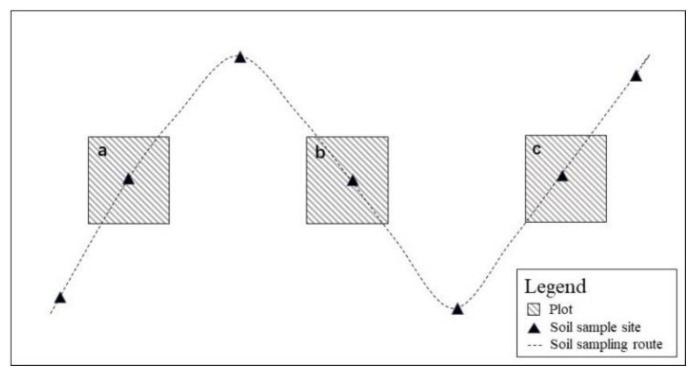
Grassland plots position (a, b, and c) and soil collected in sampling plots.

There were one hundred and seventy-one herb plots (1 m × 1 m) from fifty-seven sampling sites in the study area (Figure 2). On each plot, we recorded the names, number, coverage, proportion of all herb species and plant height of each herb. The vegetation coverage was captured by Canon fisheye lens camera and processed using ArcGIS 10.6 to get the total coverage and dominant species coverage. After the investigation, ten well-lit and developed leaves were collected from dominant species for measuring the leaf thickness, area, and dry leaf weight of the species (Table S1). The remaining leaves were taken back to the laboratory for chemical element determination after drying.

Soil samples from the fifty-seven sites were also obtained at 11:00 a.m. to 15:00 p.m. from 29 July to 31 July 2020. Seven points were selected along an S-shape line in each plot (Figure 2). The sampling was collected from 0–30 cm and mixed. Then, the soil samples were required to pass a 2 mm sieve to remove impurities, the samples were taken back to the laboratory for subsequent analysis. Soil bulk density and soil water content were obtained from three samples along the diagonal in each grassland plot, using a cylindrical metal sampler (100 mm^2^) [19].

The measurement of leaf traits was mainly carried out according to the literature [20,21]. A scanner was used to obtain the leaf area (10 replicates) and a vernier card was used to measure and record the leaf thickness. Then, the measured leaves were put into envelopes and the dry matter content of the leaves was obtained by drying method. The soil bulk density was determined by soil–core method. The SWC was calculated as the ratio of soil water mass to oven-dry weight [22,23]. The organic matter was assayed by dichromic oxidation method. The total nitrogen content was measured by Kjeldahl method using a FOSS Kjeltec 8400 Analyzer Unit (FOSS, Hillerod, Denmark) [24]. The total phosphorus was digested by H_2_SO_4_-HCIO_4_ and measured by spectrophotometer [25]. Total carbon was measured from 1 mm screened samples using Liaui TOC II analyzer (ELMENTAR, Langenselbold, Germany).

### 2.3. Variables

The SEM analysis method is a comprehensive technique that uses covariance matrix to analyze the relationships in multivariate data and identify the causality between observed variables and latent variables. In this study, we explained the relationships between the various influencing factors that pertain to plant functional traits by using latent variables and observational variables. The PLS-SEM was constructed by selecting three explanatory latent variables (topographic conditions, soil properties and vegetation structure) and one latent dependent variable (plant functional traits). Six explanatory observation variables were used to represent the latent variable characteristics of soil, namely soil organic matter content (SOM), soil water content (SWC), soil bulk density (BD), maximum soil water content (MWC), soil total phosphorus content (TP) and soil total nitrogen content (TN). Four topographic variables, namely altitude, slope, slope position and aspect, were chosen due to their effect on the hydrothermal conditions of sampling sites. In order to ensure the integrity of the vegetation diversity information, research needs of the integration of multiple levels and multiple diversity indexes [26]. We selected five different vegetation diversity indexes and two features as latent variables to illustrate vegetation structure, including the total number of plants (N), plants species index (S), Shannon–Wiener index (SHA), Simpson index (SIM), Margalef index (MAR) and Gleason index (GLE). Finally, 12 indexes, including leaf thickness, leaf dry weight, organic matter of leaves, total nitrogen content, total phosphorus content, nitrogen-to-phosphorus ratio, leaf tissue density, leaf area, specific leaf area, vegetation coverage, average plant height and ratio of dominant species were used to represent the observed variables of plant functional traits (Tables S2 and S3).

### 2.4. Calculation of Variables

The calculation and treatment of some specific variables were as follows:

(1) Margalef species richness index (MAR)

$$\mathrm{MAR}=\frac{S-1}{\ln N}$$

where S is the total number of species in the community and N is the total number of individuals of all observed species.

(2) Shannon diversity index (SHA)

$$\mathrm{SHA}=-\sum^{\;}p_{i}\ln p_{i}\;\mathrm{and}\;p_{i}=\frac{N_{i}}{N}$$

where *p_i_* is the proportion of the number of species i to the total number.

(3) Simpson diversity index (SIM)

$$S\mathrm{IM}=1-\sum^{\;}p_{i}{}^{2}\;\mathrm{and}\;p_{i}=\frac{N_{i}}{N}$$

where p_i_ is the proportion of the number of species i to the total number.

(4) Pielou’s evenness index (PIE)

$$\mathrm{PIE}=\frac{N\left(N-1\right)}{\sum^{\;}N_{i}\left(N_{i}-1\right)}$$

where N_i_ is the number of individuals of species i and N is the sum of individuals of all species in the community.

(5) Gleason richness index (GLE)

$$\mathrm{GLE}=\frac{S}{\ln A}$$

where A is the total area investigated and S is the total number of species in the community.

(6) Community weighted means (CWM)

The functional characteristics of plant communities were characterized by community weighting method [27]. CWM was calculated based on the plant functional traits of each species in the plots. In addition, the relative above-ground biomass (AGB) of each species in the plot was weighted. Additionally, the CWM mainly reflects the attribute and strategy of dominant species in the community [28,29]. The *CWM* units are the same as functional traits units involved in the calculation. The formula is as follows:

$$\mathrm{CWM}=\sum_{i=1}^{n}{\mathrm{trait}}_{i}\times P_{i}$$

where trait_i_ is the plant functional trait value of species i, P_i_ is the relative AGB of species i in the plot, and n is the number of species in the plot.

### 2.5. Selection of Variables

In the research, RF algorithm was used to filter important dependent variables to reduce the latent redundancy of dependent variables [30]. RF is an ensemble algorithm that classifies by voting on multiple unbiased classifier decision trees [31,32]. The algorithm was based on the Boruta package in R 4.1.2. The Boruta feature selection, providing important values to indicate whether features are important or not, which are obtained by mixing original attribute values between objects [33].

Plant functional traits can be reflected by multiple indices with different correlations with explanatory observed variables. Therefore, we used a random forest algorithm to screen for the indices of plant functional traits that were more correlated with explanatory observed variables. The important values of 12 plant functional traits indexes were calculated using 17 explanatory observational variables. All the plant functional traits indexes were used to classify the explanatory observed variables and an important classification value was obtained. The results for each important value were summarized and the functional trait indicators that showed statistical significance in a classification were voted on. Such voting was conducted to select more relevant and key functional traits. Random forest and voting results were used as input parameters to support latent dependent variables in the PLS-SEM model. In our research, confidence levels and maximum runs of RF algorithm were set as 0.01 and 100, respectively [19].

### 2.6. Establish of Preliminary Model and Paths Determination

Structural equation model (SEM) is a method to establish, estimate and test causality models, which contain both observable variables and latent variables that cannot be directly observed [34]. It is mainly used in PLS-SEM and structural equation modeling based on covariance. Compared with CB-PLS, which focuses on parameter evaluation, PLS-SEM has a more accurate prediction accuracy [35]. In exploratory research, we should focus on PLS-SEM when the relationship between variables is complex and unclear [36]. In addition, according to the research, when the sample number is limited and does not follow the normal distribution, PLS has a wider tolerance than CB [37,38]. Therefore, PLS-SEM was chosen in the research.

**Figure 3:**
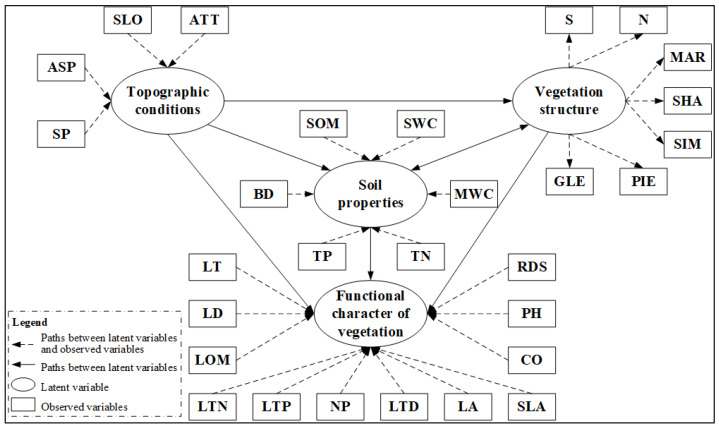
A PLS-SEM model was established to show the relationship between latent variables. The arrow represents the influence of the weight of the latent variable or observed variable on the latent variable. The ovals represent the latent variables; the rectangles represent the observed variables. SLO, slope; ALT, altitude; ASP, aspect; SP, slope position; MAR, Margalef species richness index; SHA, Shannon diversity index; SIM, Simpson diversity index; PIE, Pielou’s evenness index; GLE, Gleason richness index; N, Total number of shrubs; S, Total shrub species; SOM, Soil organic matter; BD, Soil bulk density; MWC, Maximum water capacity; TP, Total phosphorus content; TN, Total nitrogen content; SWC, Soil water content; LT, Blade thickness; LD, Leaf dry weight; LOM, Organic matter of leaves; LTN, Total nitrogen content; LTP, Total nitrogen content; NP, Nitrogen-to-phosphorus ratio; LTD, Leaf tissue density; LA, Leaf area; SLA, Specific leaf area; CO, Vegetation coverage; PH, Plant height; RDS, Ratio of dominant species.

There were three causal hypothesis to establish the preliminary model: (a) The variables of topographic conditions, soil properties and vegetation structure directly influenced dependent variables of plant functional traits; (b) The latent variables of topographic conditions indirectly impacted the latent variables of plant functional traits by influencing hydrothermal conditions, species distribution and ecological processes; (c) Vegetation and soil influence each other. The latent variables of vegetation structure indirectly impacted the latent variables of plant functional traits by influencing the latent variables of soil properties, and the latent variable of soil properties can also affect the functional character of vegetation by improving the vegetation structure (Figure 3).

To improve the reliability of model, correlation tests were carried out on different latent variables. We used CCA analysis to correlate each set of variables. Seventeen explanatory observed variables were divided into three groups of latent variables. Then, six pairs of CCA were calculated for each variable in pairs. The secondary PLS-SEM is determined by analyzing the significance of six sets of data and eliminating non-significant paths in the model. The CCA was carried out using the R package, Vegan.

### 2.7. Evaluation of PLS-SEM

We carried out factor analysis and path analysis in PLS-SEM after obtaining the results of RF and CCA analysis. In the final structural equation model, compound reliability values (CR) and average variance extraction values (AVE) are used to evaluate the structural reliability and internal model validity of SEM. The CR value > 0.7 indicates good internal consistency and reliability of the model [39]. The AVE value > 0.5 indicates that the model fits well and converges effectively. The identification validity should be evaluated with a matrix, according to the Fornell–Larcker criterion. The square root of the AVE should be greater than the relevant value of other variables [40]. We performed a variance inflation factor (VIF) analysis to avoid multicollinearity, among observing variables [41]. To avoid model misjudgment, standardized root means square residual (SRMR) was used as a fitting measure. Bootstrap programs were used to obtain T-statistics for significance tests of structural paths in PLS-SEM [42]. The path coefficient is significant when the *p*-value < 0.05 [36]. In this work, the maximum number of iterations of the PLS algorithm was set to 5000. The threshold value, which determines the maximum of the difference of the external weight, is set to 10^−7^. In addition, the number of guide sub-samples was set as 10,000 and the significance level of guide was set as 0.05 to ensure reliability. Smart PLS 3 was used for all PLS-SEM statistical analyses.

## 3. Results

Fifty-one species and 10,899 individuals of herbs were observed in 171 plots from 57 sites. *Lespedeza davurica* was the most common herb observed at 57 sites, followed by *Setaria viridis* (L.) *Beauv* at 25 sites. The twelve dependent observed variables used to explain plant functional traits were as follows: LT:0.085–0.412; LH:5.74–60.261; LOM:404.453–516.743; LTN:18.981–71.337; LTP:1.296–6.457; NP:8.078–33.559; LTD:2.423–21.617; LV:0.0655–11.479; SLA:2.994–21.071; CO:0.477–0.977; PH:10.15–55; PDS:0.4–0.975.

### 3.1. Observed Variables of Plant Functional Traits

**Figure 4:**
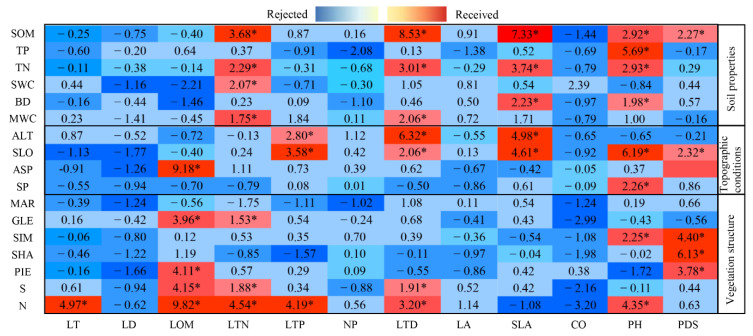
The RF is adopted for factor significance, where the red part represents acceptance and the blue part represents rejection, and * represents the significance of explanatory observation variables selected due to observation variables. SLO, Slope; ALT, Altitude; ASP, Aspect; SP, Slope position; MAR, Margalef species richness index; SHA, Shannon diversity index; SIM, Simpson diversity index; PIE, Pielou’s evenness index; GLE, Gleason richness index; N, Total number of shrubs; S, Total shrub species; SOM, Soil organic matter; BD, Soil bulk density; MWC, Maximum water capacity; TP, Total phosphorus content; TN, Total nitrogen content; SWC, Soil water content; LT, Blade thickness; LD, Leaf dry weight; LOM, organic matter of leaves; LTN, Total nitrogen content; LTP, Total nitrogen content; NP, Nitrogen-to-phosphorus ratio; LTD, Leaf tissue density; LA, Leaf area; SLA, Specific leaf area; CO, Vegetation coverage; PH, Plant height; RDS, Ratio of dominant species.

Seventeen explanatory observation variables among soil properties, vegetation structure and topographic conditions were used to classify 12 causal observation variables by RF, considering the contribution of dependent variables to explanatory variables as important values. Based on the regression analyses, we obtained 17 important values (Figure 4). The importance values of N and SOM groups showed the best results, and only four values were rejected in the group. CO was rejected by both N and SOM groups, and their importance values were −3.20 and −1.44. The importance values in MAR all indicate low importance, and all variables in this group are rejected. Eleven, fourteen and sixteen importance values are derived from topographic conditions, soil properties and diversity, respectively.

We ranked the 12 dependent variables by the 42 importance values obtained. In descending order, the results are as follows: PH (eight votes) < LTD and LTN (seven votes each) < PDS (six votes) < LOSM and SLA (five votes each) < LTP (three votes) < LT (one vote) < LD, NP, LA and CO (zero votes). PH received the highest number of votes, which were based on soil properties. LTN received four votes from soil properties, three votes from diversity, and zero votes from topographic conditions; LOM received four votes from diversity and one vote from topographic conditions; and SLA received zero votes from diversity. PH, RDS and LTD obtained votes from three latent variables, respectively, while LD, NP, LA and CO did not obtain any votes. According to the total number of votes for each dependent variable, six variables—PH, PDS, LTN, LTD, LOM and SLA—were chosen as observed variables of plant functional traits in PLS-SEM.

### 3.2. Possible Path and Model

CCA analysis showed that the relationship between topographic conditions and soil properties was not statistically significant. This may be because topographic conditions have little direct effect on the underlying variables of soil properties. The relations between vegetation structure and plant functional traits were highly statistically significant, which was consistent with the interaction between vegetation structure and functional traits. In addition, CCA results showed significant correlation between vegetation structure vs. soil properties, topographic conditions vs. vegetation structure, topographic conditions vs. plant functional traits and soil properties vs. plant functional traits. These results show the interaction among the vegetation structure, soil properties, topographic conditions and plant functional traits (Table 1).

**Figure 5:**
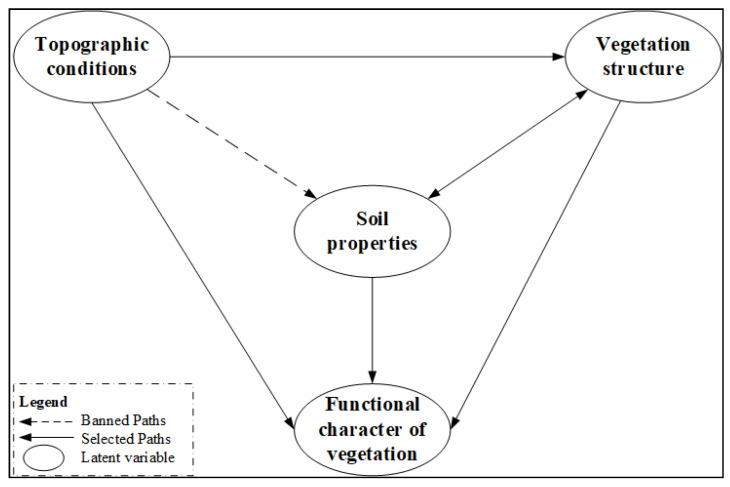
PLS-SEM based on CCA.

If CCA results between the two sets of variables are not significant, we conclude there is no latent path between the two pairs in model. Therefore, we preliminarily determined the path of the PLS-SEM based on CCA results of different variable groups by eliminating a path from topographic conditions to soil properties. The following figure describes the optimized secondary structure equation model (Figure 5).

### 3.3. Model Fit

**Figure 6:**
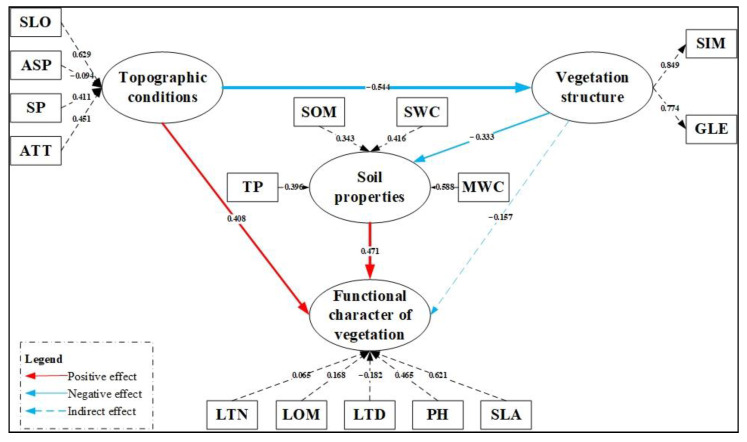
The final SEM describing the relationship between variables. The red arrow indicates positive impact and the blue indicates negative impact; the thickness of the arrow indicates the weight.

The optimal model diagram is shown below (Figure 6). In this model, the CR value is 0.795, indicating that the model has good precision and interpretability. The AVE (value = 0.660) shows that the SEM finally obtained has good fitting convergence. Identification validity was assessed through the Fornell–Larcker standard matrix. AVE square root of plant functional traits was 0.812 in the matrix, which is greater than its correlation with other variables. VIF values of all variables were less than three. The SRMR value of 0.093 is acceptable in PLS-SEM.

### 3.4. Evaluation of Influencing Factors of Plant Functional Traits

In the final PLS-SEM, the results indicate the weight of each observed variable among potential variables. The weights of PH and SLA were 0.465 and 0.621, indicating that they contributed 94.9% to latent variables of plant functional traits. For latent variables of plant species diversity, the weights of SIM and GLE were 0.849 and 0.774, respectively. SLO had the highest contribution value of latent quality variable, which was 0.629. ASP has the lowest contribution value of −0.094. The contribution rates of SOM, TP, SWC and MWC to soil properties were 0.343, 0.396, 0.416 and 0.588, respectively; they have similar contribution rates.

The bootstrapping results demonstrated an acceptable *t*-test and *p*-value, which implies that the four total effects of the final model are significant. The effects of topographic conditions on plant functional traits (*t* = 3.245, *p* < 0.01), topographic conditions on plant diversity (*t* = 5.324, *p* < 0.001), plant diversity on soil properties (*t* = 2.214, *p* < 0.05) and soil properties on plant functional traits (*t* = 3.593, *p* < 0.001) were 0. 408, −0.544, −0.333 and 0.471, respectively. In addition, the indirect effect of latent variables of plant diversity on latent variables of plant functional traits (*t* = 1.967, *p* < 0.05) was −0.157, which was acceptable according to the bootstrapping results. Finally, the remaining paths (from soil properties to vegetation structure) in the prediction model (Figure 5) were deleted because their *p* > 0.05 and would affect the overall effect of the model.

## 4. Discussion

### 4.1. Results of Screening Plant Functional Traits by RF

According to the results of RF, there are finally five indicators that get high votes, they are LTN, LOM, LTD, PH and SLA. PH received the most votes, which was mostly from soil properties. Soil is the most important factor affecting plant growth, and TP is the highest score among the four votes obtained through soil properties. The reason is that phosphorus is an indispensable factor for plant growth, which can promote the rapid growth of roots and aboveground parts and improve the ability of plants to adapt to external environmental conditions [43]. Secondly, LTN and LTD both obtained the majority of seven votes from soil properties and vegetation structure. Soil can provide nutrients for plants, and reasonable vegetation structure can provide good hydrothermal conditions for plants, LOM and SLA get higher votes for the same reason. Finally, LT received only one vote from vegetation structure, LT did not get the topographical status vote because the study area was located in SWDP, and many engineering measures changed the small topographical area of the watershed, resulting in a low impact of topography on LT. The reason for not obtaining soil voting is that the change of soil properties has limited influence on LT. As an important leaf shape component, LT is mainly affected by environmental factors, especially light intensity, which is the reason why LT can obtain vegetation structure voting.

### 4.2. Direct Effects of Topographic Conditions on Vegetation Structure and Plant Functional Traits

In the research, the direct effects of topographic conditions on plant diversity and plant functional traits [36] were evaluated. The results showed that the effects of topographic conditions on plant diversity and functional traits were mainly reflected by the differences in altitude (ALT), slope aspect (ASP) and slope position (SP), as well as the different water, heat, and light conditions, thus, leading to the formation of life strategies with different combinations of functional traits in plants. Some scholars also confirmed the same problem, the study showed that topographic factors strongly influenced the pattern of community, ecosystem, and landscape, thus, affecting plant diversity (some studies pointed out that topographic factors were the main factor, limiting the change of vegetation distribution in the loess hilly region) [44].

According to the results of PLS-SEM, topographic conditions have a negative impact on vegetation structure. SLO index had the greatest negative impact on vegetation structure. Perhaps the main reason is that with the increase in slope, due to the special natural geographical conditions of the Loess Plateau gully region, the soil required to form surface runoff when the rainfall intensity is beyond a certain range cannot effectively replenish groundwater, and thus cannot form a soil dry layer of a certain depth, eventually affecting the plant growth and reducing the plant community diversity. In the model, SP and ALT have similar contributions to vegetation structure, The main reason is that they share a similar principle: water and heat change with height, which ultimately affects the structure of the vegetation [45,46]. Finally, slope aspect had the least impact on plant diversity. Slope aspect mainly affected the light received by plants, but in the Loess Plateau region, regardless of the slope aspect, the plant received sufficient light, which resulted in a small impact of slope aspect factors on plant diversity [47].

According to the results of our research in the SWDP, the variation of SLO degree significantly affects plant functional characteristics [48]. The possible reason is that the plant growth is mainly restricted by water condition, and the change of the slope degree affects the slope surface runoff and soil erosion intensity and indirectly leads to differences in soil nutrient and moisture [49]. A study from Chinese scholars also showed that the changes of certain functional traits in plants were mainly determined by slope changes and plant functional traits gradually changed with the increase in slope value [50]. Altitude is another major variable in determining the functional traits of vegetation, which affects the temperature, water and carbon dioxide required for plant growth [51]. In addition, our results suggested that slope position is also critical to the latent variable, affecting plant functional traits. These results are consistent with the report, which showed that slope position has a significant impact on the vegetation at microtopography [52]. The main reason for this result is that the poor water and fertilizer retention ability of the upper slope position causes a large amount of water to accumulate at the bottom of the slope, resulting in significant differences in water and fertilizer conditions at different slope positions [53]. At the same time, the temperature at the bottom of the slope was higher than that at the top due to the geographical advantage, and the water and heat were locally redistributed due to the microtopography, which ultimately led to the differences in plant diversity and functional traits [54]. In this study, slope aspect contributed very little to plant functional traits (especially LTN), which was inconsistent with the results of Bennie et al. [55]. This may be because the slope aspect has little influence on soil moisture and heat in the Loess Plateau due to the low slopes and extreme drought [56].

### 4.3. Direct Effects of Soil Properties on Plant Functional Traits

Based on the results of model, in addition to topographic conditions, soil properties also directly affect the plant functional traits [57]. Some scholars also reported similar results, pointing out the availability of underground nutrients plays a key role in plant growth [58].

For target variables, our results indicate MWC and SWC are the main factors affecting plant functional traits. The biggest contribution of MWC and SWC is mainly because area is located in the Loess Plateau, where soil water retention is poor, precipitation is low and soil erosion is serious, so water is the main factor limiting plant functional traits [59]. There are other studies [60] reported that changes in plant functional traits are mainly influenced by MWC and SWC. In addition, our study showed that TN is also critical to latent variables of plant functional traits, which is consistent with Chinese scholars findings that phosphorus is an important factor, affecting plant traits in arid regions [61]. In this study, SOM contribution is 0.343. Organic matter is one of the main sources of plant nutrition, which can promote plant growth and have a great impact on plant functional traits [62]. According to the study along nutrient gradients on a provincial scale, SOM is proportional to SLA LTN, LTP, etc. [63].

### 4.4. Indirect Effect of Vegetation Structure on Plant Functional Traits

The model shows that vegetation structure (SIM and GLE) directly affect soil properties and indirectly affect plant functional traits (through soil properties), which showed there is no evidence for a direct causal correlation of species diversity and plant functional traits.

Vegetation structure has a direct negative impact on soil properties, which may be due to sub-standard soil in the Loess Plateau due to low soil moisture and nutrient content; the lack of soil nutrient accumulation and recruitment was due to excessive uptake of soil nutrients in species-rich plots. Additionally, the study area will have heavy rain that produces surface runoff and leaching from July to September, which further affects soil properties. SIM index has a great influence on soil properties. The important reason is that SIM index is an index indicating species dominance. Different species require different nutrients. Plots with high dominance indicate that the proportion of a certain species in the area is too high, which may lead to excessive consumption of certain elements in the soil and eventually have a negative impact on soil properties. For example, studies have shown that cropland (a single type of land) reduces soil organic matter content by 40% and has the worst soil structure, compared to forest and grassland [64]. Second, the GLE index of species richness based on plot area also had a negative impact on soil properties. When the plot area is fixed, there is fierce competition among vegetation in the plot with the increase in SIM index. Soil moisture is the biggest limiting factor for vegetation growth in the loess hilly–gully region. The competition between vegetation will accelerate the consumption of soil water and eventually affect soil properties. For example, the influence of vegetation structure on the spatial variation of soil’s physical and chemical properties is sometimes even more significant than that of climate factors [65]. Finally, there are two main reasons why other vegetation diversity is not used in the model. On the one hand, RF results showed that PIE, MAR and SHA had low correlations with selected plant functional traits. On the other hand, N and S indexes have collinearity with SIM and GLE in PLS-SEM. Therefore, the SIM and GLE indices were finally selected.

According to our results, plant functional traits were significantly affected by the composition and distribution of herbaceous layers [66]. Vegetation structure have a direct impact on soil properties, and the vegetation structure index can also indirectly reflect the strength of inter-vegetation competitiveness [67]. For the target variables, the model results showed that the SLA index of plant functional traits was indirect impacted by vegetation structure. SLA is a functional trait that represents the interaction between plants and the environment [68]. In the same community, the specific leaf area of plants was larger when light was decreased. When the species diversity is high, the competition among plants will lead to the lack of light for some plants, thus, affecting SLA index [69]. The change of species diversity also had a significant impact on PH, mainly because of the high light intensity and hot climate in the Loess Plateau region, but the shortage of water resources and soil nutrients. The competition among species is mainly through the root system to compete for soil water and nutrients, while the aboveground part mainly uses conservative strategies to reduce light exposure. For example, dwarf plants are more conducive to growth in the Loess Plateau. The research of scholars has also proved this point. Vegetation on the Loess Plateau generally has developed roots to obtain soil water and nutrients, while the aboveground parts are generally short [70]. In addition, changes in species diversity had a certain impact on LTN and LOM indices, and the lack of soil nutrient accumulation and recruitment was due to the excessive uptake of soil nutrients in species-rich plots. Additionally, the study area will have heavy rains that produce surface runoff and leaching from July to September, leading to a decrease in soil nutrient content, and ultimately a comprehensive impact on LTN and LOM indicators [71]. Finally, the results of PLS-SEM showed that the change of species diversity would affect the LTD; in order to adapt to the harsh living environment, plants will put more dry matter synthesized by plants into the structure of leaves (LTD increases) and improve the resistance to drought environment by increasing the distance of internal water diffusion to leaves [72].

## 5. Conclusions

Quantitative description of the relationship between the factors that may affect plant functional traits is very important for understanding the heterogeneity of vegetation habitats in Xindian National Soil and Water Conservation Demonstration Park, which can provide new insights for vegetation management and restoration in soil and water conservation area. For example, we should pay attention to vegetation structure in the future measures; reasonable vegetation structure can play a greater role in the same terrain and soil conditions. In the research, PLS-SEM was built to quantify the influence between influencing drivers on grassland vegetation functional traits, and the results showed that: (1) Topographic conditions and plant diversity were the main factors affecting plant functional traits. (2) The effect of topographic conditions on latent variables of soil properties was limited, although some topographic conditions variables were strongly correlated with it. (3) Species diversity indirectly affects latent variables of plant functional traits by influencing soil properties. In general, our results show that topographic conditions and soil properties are still the most important influencing factors, although other factors will also have an impact on the functional characteristics of vegetation; in the future, park management should continue to adhere to the policy of engineering measures in the main, with vegetation measures as the auxiliary. Finally, the results of the model are relatively reasonable for the short-term mechanism, but the time scale of the existence of the ecosystem is longer. In the future, we can continue to study SWDP to obtain the long-term mechanism and causal relationship between the components of the ecosystem in SWDP.

## Figures and Tables

**Table 1:** The significance of paths between latent variables.

Latent Variables Group	Canonical Correlations 1	Eigenvalue	*p*-Value	Wilk’s	DF
Vegetation structure vs. Soil properties	0.660	0.771	0.028 *	0.285	42
Topographic conditions vs. Vegetation structure	0.651	0.737	0.042 *	0.430	28
Topographic conditions vs. Soil properties	0.551	0.435	0.101	0.518	24
Vegetation structure vs. Functional character of vegetation	0.612	0.600	0.002 **	0.226	42
Topographic conditions vs. Functional character of vegetation	0.592	0.540	0.024 *	0.457	24
Soil properties vs. Functional character of vegetation	0.668	0.808	0.012 *	0.309	36

* indicates *p* < 0.05; ** indicates *p* < 0.01.

## Data Availability

All available data can be obtained by contacting the corresponding author.

## Supplementary Materials

The following supporting information can be downloaded at: https://www.mdpi.com/article/10.3390/plants11212891/s1, Figure S1: Slope aspect map, slope map and elevation map of the study area; Table S1: Dominant species information; Table S2: Details of variables used in the research; Table S3: The data details.
