# Supplementary material for: Agents Affecting the Plant Functional Traits in National Soil and Water Conservation Demonstration Park (China)
Source: Plants (Basel). 2022 Oct 28;11(21):2891. doi: 10.3390/plants11212891 (PMC9657439; doi:10.3390/plants11212891)
Supplement: Supplementary file 1 [file plants-11-02891-s001.zip › plants-1950597-supplementary.pdf]

plants

MDPI

Supporting information

Supplement Figures:

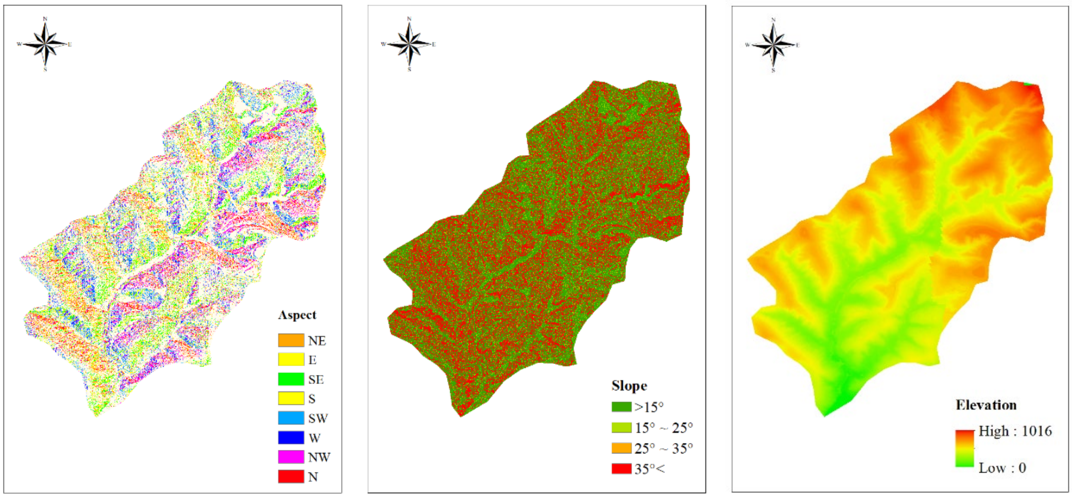

Figure S1. Slope aspect map, slope map and elevation map of the study area.

Supplement Tables:

Table S1. Dominant species information.

Family	Life form	Frequency	Mean cover	Min cover	Max cover
Asteraceae Bercht. & J. Presl	Herbs perennial	25	0.197	0.07	0.46
Valerianaceae	Herbs perennial	2	0.265	0.16	0.37
Poaceae	Herbs perennial	18	0.217	0.06	0.42
Leguminosae sp.	Herbaceous subshrubs	73	0.362	0.12	0.72
Poaceae	Herbs perennial	22	0.237	0.04	0.5
Asclepiadaceae	Herbs perennial	9	0.184	0.08	0.35
Leguminosae sp.	Small shrubs	25	0.386	0.14	0.6
Poaceae	Herbs perennial	1	0.07	0.07	0.07
Plumbaginaceae Juss.	Herbs perennial	35	0.341	0.1	0.78
Asteraceae Bercht. & J. Presl	Herbs perennial	1	0.29	0.29	0.29
Euphorbiaceae	Annual herb	1	0.06	0.06	0.06
Papilionaceae	Herbs perennial	1	0.25	0.25	0.25
Poaceae	Annual herb	51	0.283	0.03	0.58
Gramineae	Herbs perennial	2	0.235	0.15	0.32
Asteraceae Bercht. & J. Presl	Annual herb	21	0.274	0.05	0.48
Chenopodiaceae	Annual herb	1	0.18	0.18	0.18
Zygophyllaceae R. Br.	Annual herb	1	0.1	0.1	0.1
Asteraceae Bercht. & J. Presl	Herbs perennial	21	0.29	0.15	0.58
Bignoniaceae Juss.	Herbs perennial	1	0.1	0.1	0.1
Asteraceae Bercht. & J. Presl	Herbs perennial	1	0.1	0.1	0.1
Cyperaceae Juss.	Herbs perennial	20	0.355	0.13	0.75
Rubiaceae Juss.	Herbs perennial	10	0.354	0.15	0.63
Solanaceae Juss.	Annual herb	5	0.33	0.18	0.42
Ranunculaceae Juss.	Herbs perennial	1	0.55	0.55	0.55
Convolvulaceae Juss.	Herbs perennial	5	0.224	0.14	0.32
Papilionaceae	Annual herb	2	0.165	0.15	0.18
Liliaceae	Herbs perennial	2	0.24	0.09	0.39
Poaceae	Herbs perennial	9	0.263	0.19	0.65
Asteraceae Bercht. & J. Presl	Herbs perennial	6	0.307	0.12	0.54
Leguminosae sp.	Herbs perennial	1	0.2	0.2	0.2
Asteraceae Bercht. & J. Presl	Herbaceous subshrubs	17	0.233	0.12	0.5
Brassicaceae	Herbs perennial	1	0.25	0.25	0.25
Poaceae	Herbs perennial	1	0.18	0.18	0.18
Chenopodiaceae	Annual herb	5	0.312	0.2	0.53

Table S2. Details of variables used in the research.

Latent Variable	Observed Variable	Abbreviation	Detail
Soil property	Soil organic matter	SOM	The content of organic matter component of soil
	Soil water content	SWC	The content of water in soil
	Soil bulk density	BD	The weight of soil in a unit volume
	Maximum water capacity	MWC	The moisture content of soil when the capillary is filled with water
	Total phosphorus content	TP	The content of total phosphorus of soil
	Total nitrogen content	TN	The content of total nitrogen of soil
Vegetation diversity	Margalef species richness index	MAR	Species richness index based on total number
	Shannon diversity index	SHA	Index of species diversity
	Simpson diversity index	SIM	Index of species dominance
	Pielou's evenness index	PIE	Index of species evenness
	Gleason richness index	GLE	Species richness index based on plot area
	Total number of shrubs	N	Total number of plant individuals
	Total shrub species	S	Total number of plant species
Site conditions	Slope	SLO	Mean angle of the site to the horizontal
	Altitude	ATT	Mean altitude of each plot
	Aspect	ASP	The direction of the projection of a slope normal onto a horizontal plan
	Slope positions	SP	Geomorphic position on the slope where the sample site is located
Functional character of vegetation	Blade thickness	LT	Blade thickness of each plot
	Leaf dry weight	LD	Leaf dry weight of each plot
	organic matter of leaves	LOM	Organic matter of leaves in each plot
	Total nitrogen content	LTN	Total nitrogen content of leaves in each plot
	Total phosphorus content	LTP	Total phosphorus content of leaves in each plot
	Nitrogen to phosphorus ratio	NP	The ratio of the amount of all nitrogen to the amount of phosphorus in each plot
	Leaf tissue density	LTD	Ratio of leaf dry weight to leaf volume
	Leaf area	LA	Leaves area of each plot
	Specific leaf area	SLA	Ratio of leaf area to dry weight in each plot
	Vegetation coverage	CO	The aggregate of all vertically projected plants onto the ground surface
	Plant height	PH	Average height of all the plants in a plot
	Ratio of dominant species	RDS	The proportion of biomass of all dominant species in the community to the total biomass

Table S3. The data details.

Index	SOM	TP	TN	SWC	BD	MWC	ALT	SLO
Average	7.61±4.24	0.59±0.12	0.41±0.21	0.15±0.08	1.25±0.12	0.40±0.09	979.04±61.15	25.19±12.23
Index	ASP	SP	MAR	GLE	SIM	SHA	PIE	S
Average	0.65±0.35	0.6±0.2	1.69±1.15	8.88±5.69	0.74±0.18	1.65±0.86	0.74±0.16	9±5
Index	N	LT	LD	LOM	LTN	LTP	NP	LTD
Average	196±134	0.16±0.26	24.93±19.23	456.2±51.8	42.3±24.68	2.70±1.54	15.98±7.92	7.87±5.51
Index	LA	SLA	CO	PH	RDS			
Average	4.04±3.39	9.84±6.85	0.77±0.23	25.53±15.45	0.80±0.2			
